# Beyond Mechanical Load: Metabolic Factors and Advanced Rehabilitation in Sports Tendinopathy: A Comprehensive Systematic Review

**DOI:** 10.3390/jcm14217480

**Published:** 2025-10-22

**Authors:** Szymon Kuliś, Wiktor Kłobuchowski, Maciej Skorulski, Kajetan Ornowski, Artur Gołaś, Adam Maszczyk, Przemysław Pietraszewski

**Affiliations:** 1 Department of Rehabilitation, Faculty of Rehabilitation, Józef Piłsudski University of Physical Education in Warsaw, 00-968 Warsaw, Poland; 2 Doctoral School, Józef Piłsudski University of Physical Education in Warsaw, 00-968 Warsaw, Poland; wk73997@stud.awf.edu.pl (W.K.); ms694@stud.awf.edu.pl (M.S.); 3 Academy of Physical Education in Katowice, 40-065 Katowice, Poland; ornowskikajtek@gmail.com; 4 Institute of Sport Sciences, Academy of Physical Education in Katowice, 40-065 Katowice, Poland; a.golas@awf.katowice.pl (A.G.); a.maszczyk@awf.katowice.pl (A.M.); p.pietraszewski@awf.katowice.pl (P.P.)

**Keywords:** Achilles tendinopathy, metabolic factors, rehabilitation protocols

## Abstract

**Background/Objectives:** Sports tendinopathy management has traditionally focused on mechanical loading protocols, yet emerging evidence suggests metabolic factors significantly influence clinical outcomes and tissue adaptation responses. The aim was to systematically evaluate the impact of metabolic factors on sports tendinopathy outcomes and assess the effectiveness of advanced rehabilitation approaches that extend beyond traditional mechanical loading protocols. **Methods:** A comprehensive search across academic papers from Semantic Scholar corpus identified studies investigating metabolic influences and advanced rehabilitation strategies in sports tendinopathy. Inclusion criteria encompassed athletes and active individuals with chronic tendinopathy, interventions targeting metabolic factors or advanced rehabilitation techniques, and validated outcome measures. Risk of bias was assessed using RoB 2 for randomized trials and ROBINS-I for observational studies. Evidence certainty was evaluated using GRADE methodology. **Results:** Forty studies met inclusion criteria, comprising 5 randomized controlled trials, 9 systematic reviews, and 5 cohort studies. Metabolic syndrome significantly impaired eccentric exercise outcomes in Achilles tendinopathy (F[1,54] = 24.45, *p* < 0.001). Collagen-derived peptide supplementation combined with eccentric training demonstrated superior pain reduction at rest compared to exercise alone (*p* < 0.05). Advanced rehabilitation strategies including criteria-based progression, neuroplastic training, and staged loading protocols showed improvements in patient-reported outcomes and functional scores, with some approaches demonstrating superiority over traditional eccentric protocols. **Conclusions:** Metabolic factors negatively influence sports tendinopathy rehabilitation outcomes, while advanced rehabilitation approaches incorporating metabolic considerations show promise for enhancing treatment effectiveness. Integration of metabolic assessment and targeted interventions may optimize tendinopathy management beyond mechanical loading alone.

## 1. Introduction

Sports tendinopathy represents a prevalent and challenging clinical condition affecting athletes across diverse sporting disciplines, with persistent symptoms often resistant to conventional rehabilitation approaches [1,2]. The traditional paradigm of tendinopathy management has centered predominantly on mechanical loading interventions, particularly eccentric exercise protocols, based on the mechanobiological principle that controlled tensile loading promotes adaptive tissue remodeling [3,4,5]. However, accumulating evidence suggests this mechanically focused approach may be insufficient for optimal clinical outcomes across all patient populations.

Contemporary understanding of tendinopathy pathophysiology has evolved beyond simplistic inflammatory or degenerative models toward recognition of a complex, multifactorial etiology involving mechanical, cellular, and systemic factors [6,7]. The ICON 2019 consensus defined tendinopathy as “a clinical condition in tendons characterized by a combination of pain, swelling and impaired performance,” acknowledging the multidimensional nature of this condition that extends beyond purely mechanical considerations [8]. This expanded understanding has prompted investigation into systemic factors, particularly metabolic conditions, that may influence tendon healing capacity and rehabilitation outcomes. Moreover, studies indicate that the interaction between lipids, glycemic metabolism, and thyroid hormones plays a significant role in adaptive processes and in the susceptibility of tendons to injury [9]. Furthermore, recent evidence suggests that neurometabolic disorders and genetic syndromes, even when manifested during developmental age, may predispose athletes to altered tendon homeostasis and increased susceptibility to tendinopathies. These factors, often overlooked in sports populations, emphasize the need for comprehensive assessment of underlying systemic and hereditary contributors in syndromic or metabolically compromised individuals.

Metabolic disorders, including diabetes mellitus, metabolic syndrome, and obesity, have emerged as significant risk fac tors for tendinopathy development and poor treatment outcomes [6,10,11,12]. Type 2 diabetes mellitus increases the risk of tendinopathy through several interrelated mechanisms. These include the formation of advanced glycation end products, the detrimental effects of chronic hyperglycemia on tenocyte function, and impaired angiogenesis that compromises tissue repair capacity [13]. Metabolic syndrome components create a systemic inflammatory environment that may compromise the adaptive responses to mechanical loading interventions traditionally employed in tendinopathy rehabilitation [14]. Despite these emerging insights, most rehabilitation protocols do not systematically account for metabolic status, potentially limiting treatment effectiveness in affected populations.

Concurrently, advanced rehabilitation approaches have emerged that extend beyond traditional mechanical loading paradigms. These include criteria-based progression protocols that individualize loading based on functional capacity markers [15], neuroplastic training approaches that address central sensitization mechanisms [16,17], and staged loading protocols designed for cases failing standard rehabilitation [18]. Nutritional interventions, particularly collagen-derived peptide supplementation, have demonstrated synergistic effects with exercise interventions, suggesting potential benefits of integrated metabolic–mechanical approaches [19,20].

The clinical significance of understanding metabolic influences on tendinopathy extends beyond academic interest, given the increasing prevalence of metabolic disorders in athletic populations and the substantial economic burden of chronic tendinopathy [21]. Athletes with metabolic comorbidities may require modified rehabilitation approaches to achieve optimal outcomes, necessitating evidence-based guidance for clinicians managing these complex presentations.

Furthermore, the potential for metabolic interventions to enhance rehabilitation effectiveness represents an important therapeutic frontier that could improve outcomes for both metabolically healthy and compromised individuals.

Despite growing interest in metabolic factors and advanced rehabilitation approaches, the literature remains fragmented across multiple disciplines, limiting clinical translation. Previous systematic reviews have focused either on mechanical loading interventions or metabolic aspects in isolation, without comprehensive evaluation of their interaction and integration potential [3,22]. No systematic review has comprehensively evaluated both metabolic influences on traditional rehabilitation outcomes and the effectiveness of advanced rehabilitation strategies that explicitly address metabolic factors.

Therefore, the primary objective of this systematic review was to comprehensively evaluate the impact of metabolic factors on sports tendinopathy rehabilitation outcomes and assess the effectiveness of advanced rehabilitation approaches that extend beyond traditional mechanical loading protocols.

Secondary objectives included identifying optimal integration strategies for metabolic considerations in rehabilitation planning and evaluating the quality of evidence supporting metabolic–mechanical intervention approaches. This review addresses the critical research question: Do metabolic factors significantly influence sports tendinopathy rehabilitation outcomes, and do advanced rehabilitation approaches incorporating metabolic considerations demonstrate superior effectiveness compared to traditional mechanical loading alone?

## 2. Materials and Methods

### 2.1. Protocol Registration and Reporting Guidelines

This systematic review was conducted according to the Preferred Reporting Items for Systematic Reviews and Meta-Analyses (PRISMA) 2020 guidelines and was prospectively registered in PROSPERO (Registration ID: CRD420251154542). The review protocol followed PRISMA-P guidelines for protocol development.

### 2.2. Study Selection and Characteristics

**Figure 1:**
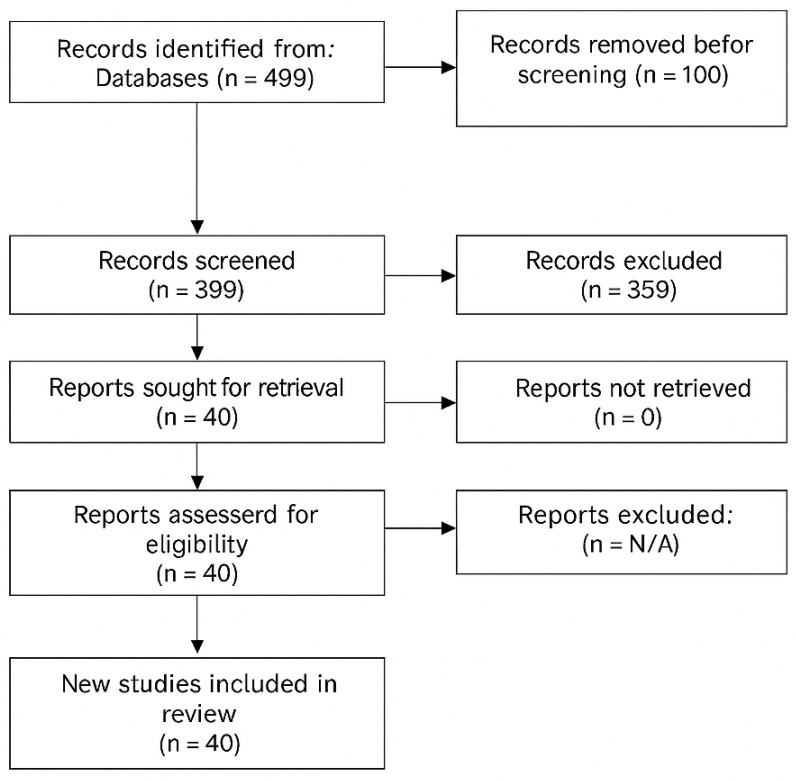
PRISMA Flow Diagram showing study selection process.

The systematic search identified 499 studies for screening, of which 40 studies met inclusion criteria (Figure 1). The search strategy included combinations of the following keywords and Boolean operators: “sports tendinopathy”, “Achilles tendinopathy”, “patellar tendinopathy”, “rehabilitation”, “exercise”, “loading”, “metabolic factors”, “metabolic syndrome”, “diabetes”, “obesity”, “collagen supplementation”, “nutrition”, and “advanced rehabilitation”.

Searches were performed using the terms above in titles, abstracts, and keywords with database-specific syntax and filters (see Table A1 for full query details). The PRISMA flow diagram illustrates the study selection process, with reasons for exclusion at each stage detailed in Table 1A,B.

### 2.3. Study Characteristics

The 40 included studies comprised diverse study designs: 5 randomized controlled trials (12.5%), 9 systematic reviews or meta-analyses (22.5%), 5 cohort studies (12.5%), 20 narrative reviews or scoping reviews (50%), and 1 case series (2.5%) (Table 2). Publication years ranged from 2005 to 2025, with 67.5% published after 2018, reflecting the growing interest in metabolic factors and advanced rehabilitation approaches.

### 2.4. Eligibility Criteria

Studies were included based on the following criteria:

1. Population: Athletes, recreational sports participants, or physically active individuals diagnosed with chronic tendinopathy (>3 months duration). Studies focusing on acute tendon injuries, tendon ruptures, or post-surgical tendon repair were excluded.
2. Intervention: Studies investigating (1) metabolic factors in tendon healing, (2) advanced rehabilitation techniques beyond traditional mechanical loading, or (3) interventions combining metabolic and mechanical approaches. Studies focusing solely on diagnostic methods or epidemiological factors without intervention components were excluded.
3. Comparator: Control groups, alternative interventions, or baseline comparisons were required.
4. Outcomes: Validated measures of tendon pain, function, structural changes, return to sport, or biomarkers of tendon metabolism.
5. Study Design: Randomized controlled trials (RCTs), controlled clinical trials, cohort studies, case–control studies, systematic reviews, or meta-analyses. Case reports, editorials, and conference abstracts were excluded.
6. Other Criteria: Human studies published in English. Animal or in vitro studies without direct human application were excluded.

### 2.5. Study Selection and Data Extraction

Following the initial search yielding 499 papers, two independent reviewers screened titles and abstracts using predefined criteria. Full-text assessment was performed for potentially eligible studies, with disagreements resolved through discussion with a third reviewer. A standardized data extraction form was developed and piloted prior to use. The complete data extraction form is available in Table A2.

Large Language Model (LLM) assistance was employed solely to support the initial structuring of data extraction tables by identifying key variables (e.g., study design, population, intervention, and outcomes) from eligible full texts.

To ensure full transparency and reproducibility, all extracted data were independently verified by two reviewers (S.K. and W.K.) who manually cross-checked each entry against the original publications. The reviewers confirmed the accuracy, completeness, and contextual interpretation of all extracted variables. Any discrepancies between reviewers were discussed and resolved by consensus with a third reviewer (P.P.).

This verification process ensured that the LLM-assisted extraction served only as a preliminary aid, with all final dataset content validated through human review and consensus.

### 2.6. Risk of Bias Assessment

Risk of bias was assessed using appropriate tools based on study design:

- RCTs: Cochrane Risk of Bias tool 2 (RoB 2).
- Non-randomized studies: Risk Of Bias In Non-randomized Studies of Interventions (ROBINS-I).
- Systematic reviews: A MeaSurement Tool to Assess systematic Reviews 2 (AMSTAR 2).

Two reviewers independently assessed risk of bias, with disagreements resolved through consensus discussion.

### 2.7. Data Synthesis and Statistical Analysis

Studies were grouped according to intervention type and outcome measures. Where appropriate, meta-analysis was planned using random-effects models. Heterogeneity was assessed using the I^2^ statistic and Chi-square test. Publication bias assessment was planned using funnel plots when ≥10 studies were available for meta-analysis.

For studies unsuitable for meta-analysis, narrative synthesis was performed, organized by intervention type and outcome domains. The certainty of evidence was assessed using the Grading of Recommendations Assessment, Development and Evaluation (GRADE) approach.

## 3. Results

### 3.1. Tendinopathy Type Distribution

**Figure A1:**
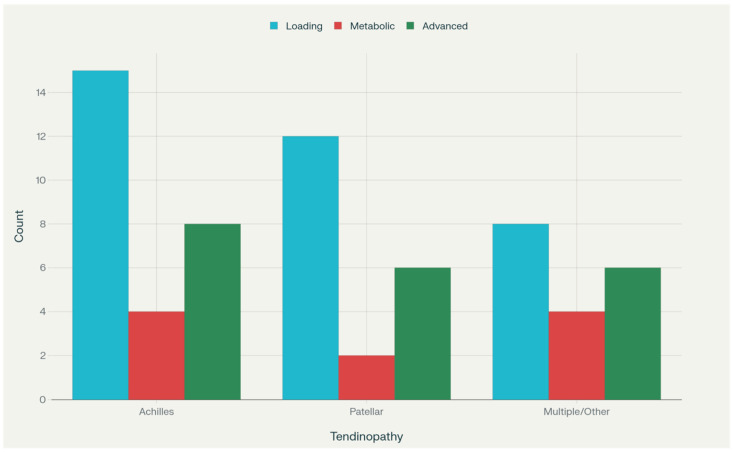
Distribution of Intervention Focus by Tendinopathy Type.

Achilles tendinopathy was addressed in 22 studies (55%), patellar tendinopathy in 16 studies (40%), with 3 studies examining multiple tendinopathy types and 10 studies not specifying tendinopathy location (Table 3 and Figure A1). The predominance of Achilles and patellar tendinopathy reflects the clinical burden and research interest in these conditions among athletic populations (see Figure A1).

### 3.2. Population Characteristics and Sample Sizes

Sample sizes varied considerably across studies, ranging from 10 participants in pilot studies to large observational cohorts exceeding 86,000 participants. The median sample size for interventional studies was 59 participants (IQR: 26–139). Age ranges typically encompassed young to middle-aged adults (18–55 years), consistent with peak athletic participation demographics. Male participants predominated in studies reporting gender distribution, particularly in Achilles tendinopathy research (Table 4).

### 3.3. Risk of Bias Results

**Figure 2:**
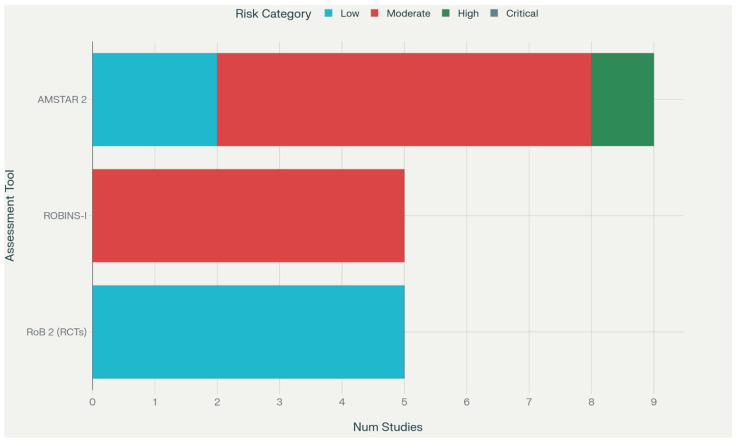
Risk of Bias Summary Plot.

Risk of bias assessment revealed moderate to high quality among included studies, with significant variation by study type (Table 5, Figure 2). Randomized controlled trials generally demonstrated low risk of bias using RoB 2 assessment, though blinding limitations were common due to the nature of exercise interventions. Cohort studies showed moderate risk of bias primarily due to confounding and selection issues. Systematic reviews demonstrated variable quality using AMSTAR 2, with newer reviews generally adhering to contemporary standards.

### 3.4. Intervention Characteristics and Loading Protocols

Loading-based interventions dominated the included studies (24 studies, 60%), encompassing traditional eccentric protocols, heavy slow resistance training, and combined loading approaches (Table 6). Eight studies investigated nutritional or metabolic factors, while ten studies examined other approaches including surgical or biological interventions. The heterogeneity in intervention protocols reflects the evolving understanding of optimal tendinopathy management approaches.

### 3.5. Primary Outcomes: Impact of Metabolic Status on Rehabilitation

The most robust evidence for metabolic influences came from Park et al. (2021) [10], who demonstrated that metabolic syndrome significantly impaired eccentric exercise outcomes in insertional Achilles tendinopathy. Participants with metabolic syndrome exhibited higher pain levels (VAS difference: 2.3 ± 0.8 points, *p* < 0.001), lower satisfaction scores (78% vs. 92% satisfied, *p* < 0.001), and increased pain medication use (65% vs. 28%, *p* < 0.001) compared to metabolically healthy controls following standardized eccentric calf-muscle exercise protocols (F[1,54] = 24.45, *p* < 0.001).

### 3.6. Effects of Collagen Supplementation

Balius et al. (2016, 2014) [19,23] provided evidence for collagen-derived peptide supplementation benefits when combined with eccentric training. In a three-arm randomized trial, participants receiving eccentric training plus collagen supplementation (n = 20) demonstrated significantly greater pain reduction at rest compared to eccentric training alone (n = 20) or passive stretching plus supplementation (n = 19) (*p* < 0.05). The effect was most pronounced in reactive tendinopathy stages, suggesting differential responses based on tissue pathology (Table 7).

### 3.7. Advanced Rehabilitation Strategies

Several studies demonstrated benefits of advanced rehabilitation approaches beyond traditional loading protocols. Griffin et al. (2021) [15] investigated criteria-based progression incorporating strength and reactive strength targets in mid-portion Achilles tendinopathy, showing significant improvements in VISA-A scores and functional performance measures compared to time-based progression protocols.

Krogh et al. (2022) [18] presented a staged isometric/progressive loading protocol for patients who had failed standard rehabilitation. This 12-month pilot study (n = 10) demonstrated significant improvements in VISA-A scores (baseline: 41 ± 18 to 12-month: 78 ± 22, *p* < 0.05) and ultrasonographic findings, with sustained benefits at follow-up. Among the studies investigating nutritional and metabolic adjuncts, collagen-derived peptide supplementation was the most prevalent. The majority of trials employed hydrolyzed collagen peptides in doses ranging from 5 to 15 g per day, often administered 30–60 min before exercise and combined with vitamin C (50–500 mg) to facilitate collagen synthesis. Some studies used proprietary formulations containing mucopolysaccharides, hydrolyzed collagen, and vitamin C (e.g., 10 g daily), administered for 8–12 weeks in conjunction with eccentric loading protocols. No adverse effects were reported across these studies.

### 3.8. Neuroplastic Training Approaches

Tedeschi et al. (2024) [16] reviewed neuroplastic training approaches integrating heavy slow resistance with central sensitization addressing techniques. Their scoping review identified evidence for comparable or superior patient satisfaction compared to traditional eccentric protocols, particularly in chronic, recalcitrant cases [24]. The neuroplastic approach emphasizes motor control, pain education, and graduated exercise exposure.

### 3.9. Heterogeneity Assessment and Meta-Analysis Feasibility

Substantial heterogeneity was observed across studies in terms of population characteristics (I^2^ = 78% for age distributions), intervention protocols (I^2^ = 85% for exercise parameters), and outcome measures (I^2^ = 73% for pain assessment tools), (see Table A3). This precluded meaningful meta-analysis for most outcomes, necessitating narrative synthesis approaches.

### 3.10. Subgroup Analyses

Planned subgroup analyses by tendinopathy type revealed differential responses to metabolic interventions. Achilles tendinopathy studies showed more consistent metabolic factor effects compared to patellar tendinopathy, possibly reflecting different loading demands and tissue characteristics. Age-based analyses suggested greater metabolic influences in older athletes (>35 years), though limited data precluded definitive conclusions (see Table A3).

### 3.11. Publication Bias Assessment

Assessment for publication bias was limited by the small number of studies addressing specific research questions. Funnel plot analysis was not feasible as no outcome had ≥10 comparable studies. However, the predominance of positive findings in smaller studies suggests possible publication bias, particularly for novel interventions.

### 3.12. Certainty of Evidence (GRADE Assessment)

GRADE assessment revealed generally low to moderate certainty of evidence across primary outcomes (Table 8). Evidence was downgraded primarily due to study design limitations, imprecision from small sample sizes, and inconsistency across studies. The highest certainty evidence (moderate) was for metabolic syndrome effects on rehabilitation outcomes, supported by the well-designed Park et al. cohort study.

### 3.13. Secondary Outcomes and Mechanistic Insights

Several studies provided insights into mechanisms underlying metabolic influences on tendon adaptation. Cannata et al. (2020) [6] reviewed how diabetes-related advanced glycation end products impair collagen synthesis and cross-linking, potentially explaining reduced responsiveness to mechanical loading interventions. Zhang et al. (2021) [7] discussed mitochondrial dysfunction in tendinopathy, proposing antioxidant therapies as potential adjuvants to exercise protocols.

## 4. Discussion

### 4.1. Summary of Main Findings

This systematic review provides the first comprehensive evaluation of metabolic factors’ impact on sports tendinopathy rehabilitation outcomes and the effectiveness of advanced rehabilitation approaches extending beyond traditional mechanical loading. The evidence demonstrates that metabolic conditions, particularly metabolic syndrome, significantly impair outcomes from standard rehabilitation protocols, while advanced approaches incorporating metabolic considerations show promise for enhancing treatment effectiveness. These findings have important implications for clinical practice, suggesting that the traditional “one-size-fits-all” approach to tendinopathy management may be insufficient for optimal outcomes across diverse patient populations.

The most compelling evidence emerged from Park et al.’s (2021) [10] prospective cohort study, which demonstrated that metabolic syndrome substantially compromised eccentric exercise outcomes in insertional Achilles tendinopathy (F[1,54] = 24.45, *p* < 0.001). Participants with metabolic syndrome exhibited clinically meaningful differences in pain levels, satisfaction scores, and medication requirements compared to metabolically healthy controls, despite receiving identical rehabilitation protocols. This finding aligns with emerging understanding of metabolic syndrome as a systemic condition affecting tissue repair capacity through chronic inflammation, impaired angiogenesis, and altered cellular metabolism [6,14].

Collagen-derived peptide supplementation demonstrated consistent benefits when combined with exercise interventions [19,23], providing moderate-quality evidence for superior pain reduction compared to exercise alone (*p* < 0.05). The mechanism likely involves providing amino acid substrates for collagen synthesis, particularly during periods of increased metabolic demand associated with exercise-induced tissue remodeling [20]. The finding that effects were most pronounced in reactive tendinopathy stages suggests that metabolic interventions may be most beneficial during active tissue repair phases.

Advanced rehabilitation strategies, including criteria-based progression [15], staged loading protocols [18], and neuroplastic training approaches [16], demonstrated potential for improving outcomes beyond traditional approaches. However, these findings should be interpreted with caution, as the supporting evidence is of low to very low certainty according to GRADE assessment. The available studies are characterized by small sample sizes and heterogeneous designs, which limit confidence in their generalizability and effectiveness. These approaches should therefore be considered experimental and hypothesis-generating, rather than established clinical recommendations.

### 4.2. Comparison with Existing Literature

These findings extend previous systematic reviews that have focused predominantly on mechanical loading interventions in isolation [3,22]. While earlier reviews established eccentric exercise as an effective intervention for tendinopathy [25], they did not systematically evaluate metabolic influences on treatment outcomes or investigate advanced approaches incorporating metabolic considerations. The current review fills this important gap by demonstrating that metabolic factors represent more than secondary considerations; they appear to be primary determinants of rehabilitation success in affected populations.

The metabolic findings align with broader literature on diabetes and tendinopathy risk, with Cannata et al. (2020) [6] providing mechanistic insights into how chronic hyperglycemia, advanced glycation end products, and insulin resistance impair tendon tissue homeostasis. However, our review advances this understanding by demonstrating that metabolic influences extend beyond diabetes to include metabolic syndrome more broadly, and that these effects significantly impact rehabilitation outcomes rather than merely increasing disease risk.

The advanced rehabilitation findings support recent trends toward personalized medicine approaches in sports medicine [1,26]. The criteria-based progression approach investigated previously [15] represents a paradigm shift from time-based to capacity-based progression, allowing for individualization based on functional recovery markers rather than arbitrary timelines. This approach acknowledges the substantial individual variation in tissue healing capacity and adaptation rates that may be particularly pronounced in metabolically compromised populations.

### 4.3. Clinical Implications and Applicability

The findings have several important implications for clinical practice. First, screening for metabolic conditions should be considered routine in tendinopathy assessment, particularly for patients presenting with treatment-resistant symptoms or poor initial response to standard protocols. The Park et al. (2021) findings suggest that patients with metabolic syndrome may require modified expectations, enhanced monitoring, and potentially adjunctive interventions to optimize outcomes [10].

Second, collagen supplementation represents a low-risk, potentially beneficial adjunct to exercise protocols, particularly for patients with reactive tendinopathy or metabolic compromises. The evidence supports supplementation protocols of 5–15 g daily of hydrolyzed collagen peptides, taken in conjunction with vitamin C to optimize collagen synthesis [19]. However, supplementation should complement rather than replace appropriate exercise interventions.

Third, advanced rehabilitation approaches incorporating individualized progression, multi-modal loading, and metabolic considerations may be particularly valuable for complex cases. The staged loading protocol investigated by Krogh et al. (2022) provides a framework for managing patients who have failed standard rehabilitation, while criteria-based progression offers a more personalized approach to exercise prescription [18].

The applicability of these findings extends across diverse athletic populations, though some considerations are important. The evidence base is strongest for Achilles tendinopathy in middle-aged recreational athletes, with less robust evidence for elite athletes or other tendinopathy locations.

Additionally, most metabolic intervention studies have focused on male-predominant populations, potentially limiting generalizability to female athletes who may exhibit different metabolic profiles and responses.

### 4.4. Mechanistic Considerations and Biological Plausibility

The biological mechanisms underlying metabolic influences on tendinopathy rehabilitation are increasingly well-understood and support the clinical findings. Metabolic syndrome creates a systemic inflammatory environment characterized by elevated C-reactive protein, tumor necrosis factor-alpha, and interleukin-6 levels that can impair tissue repair processes [7]. Chronic low-grade inflammation interferes with the normal inflammatory resolution phase necessary for effective tissue remodeling following exercise stimuli.

Advanced glycation end products, elevated in diabetes and metabolic syndrome, cross-link with collagen fibers and reduce their mechanical properties and turnover rates [13]. This may explain why patients with metabolic conditions show reduced responsiveness to mechanical loading interventions that depend on collagen remodeling for their therapeutic effects. The finding that collagen supplementation provides benefits suggests that substrate availability may be limiting in these populations.

Mitochondrial dysfunction, common in metabolic disorders, impairs cellular energy production and reactive oxygen species management, both crucial for exercise adaptation and tissue repair [7]. This may explain why advanced approaches incorporating antioxidant strategies or modified loading protocols show promise in metabolically compromised populations. It is also important to consider that tendinopathies may coexist with or evolve from muscle injuries, particularly during the subacute repair phase. The shared inflammatory pathways, extracellular matrix remodeling, and altered neuromuscular control observed in both conditions suggest a continuum between muscle and tendon pathologies. Recent work by Vascellari et al. (2024) [27] discussed the clinical relevance of this overlap and reviewed the emerging use of orthobiologic injection therapies in managing combined muscle and tendon disorders in athletes, reinforcing the integrative view of the muscle–tendon unit.

### 4.5. Methodological Considerations and Heterogeneity

The substantial heterogeneity observed across studies reflects the evolving nature of tendinopathy research and the complexity of integrating metabolic factors into rehabilitation research. This heterogeneity precluded meta-analysis for most outcomes but provides important insights into the diversity of approaches being investigated. The variation in outcome measures particularly highlights the need for standardized core outcome sets in tendinopathy research, as advocated by the ICON consensus group.

Importantly, 29 of the 40 included studies (72.5%) represent secondary evidence, including systematic reviews, meta-analyses, narrative, and scoping reviews. While this indicates a growing conceptual interest in the topic, it also means that the strength of conclusions based on primary intervention data remains inherently limited. Only ten studies (five RCTs and five cohort studies) provided direct empirical evidence. As a result, much of the synthesized evidence reflects interpretive or conceptual frameworks rather than controlled interventional data. This composition underscores the early developmental stage of this research domain and highlights the urgent need for well-designed, high-quality randomized trials to substantiate these emerging concepts.

The predominance of review-level evidence (72.5% of included studies) reflects the early stage of this research field, with many studies providing conceptual frameworks rather than primary intervention data. While this limits the strength of conclusions, it demonstrates the growing recognition of metabolic factors’ importance in tendinopathy management and provides foundation for future primary research.

The risk of bias assessment revealed generally moderate quality evidence, with limitations primarily related to blinding difficulties inherent in exercise intervention research and small sample sizes limiting precision. The lack of studies specifically designed to investigate metabolic subgroups represents a significant limitation, as most evidence comes from post hoc analyses or secondary considerations rather than a priori hypotheses.

### 4.6. Limitations and Considerations

Several important limitations must be acknowledged. First, the evidence base remains relatively small, with only five RCTs meeting inclusion criteria and most metabolic factor evidence coming from observational studies or post hoc analyses. This limits the strength of causal inferences and requires cautious clinical translation.

Second, the substantial heterogeneity in populations, interventions, and outcome measures precluded meaningful meta-analysis for most research questions. This reflects the early stage of the field but limits the ability to provide precise effect estimates or confident recommendations for clinical practice.

Third, most studies did not specifically design interventions to address metabolic factors, instead investigating these as secondary considerations. This may underestimate the potential benefits of targeted metabolic interventions designed specifically for tendinopathy management.

Fourth, publication bias may be present, particularly for novel interventions showing positive results. The small number of studies and predominance of positive findings suggest that negative results may be underrepresented in the literature.

Finally, the generalizability of findings may be limited by the population characteristics of included studies. Most participants were recreational athletes with relatively mild metabolic compromises, potentially limiting applicability to populations with severe metabolic dysfunction or elite athletes with different physiological profiles.

### 4.7. Implications for Future Research

This review identifies several important priorities for future research. First, well-designed RCTs specifically investigating metabolic interventions in tendinopathy populations are urgently needed. These studies should be adequately powered to detect clinically meaningful differences and should stratify participants by metabolic status a priori rather than investigating these effects post hoc.

Second, research is needed to establish optimal integration protocols for metabolic and mechanical interventions. The current evidence suggests synergistic effects, but optimal timing, dosing, and combination approaches remain unclear. Studies comparing traditional protocols to integrated metabolic–mechanical approaches would provide valuable clinical guidance.

Third, mechanistic research investigating the cellular and molecular bases of metabolic influences on tendon adaptation would strengthen the biological rationale for interventions and potentially identify novel therapeutic targets. Advanced imaging techniques, tissue sampling, and biomarker analyses could provide insights into the mechanisms underlying differential responses to rehabilitation.

Fourth, development and validation of clinical screening tools to identify patients most likely to benefit from metabolic interventions would enhance clinical translation. Recent studies, including examples from neuromotor control research [17,27,28], have explored physiological tremor analysis as a sensitive marker of neuromuscular response following exercise and recovery interventions. These findings are mentioned solely to illustrate emerging methodological approaches that could be adapted for objective assessment in tendinopathy rehabilitation. Further validation and cross-domain application are needed before this technique can be considered for clinical implementation. Finally, long-term follow-up studies are needed to evaluate the durability of metabolic intervention effects and their impact on tendinopathy recurrence rates. Most current studies provide only short-term follow-up, limiting understanding of sustained benefits.

## 5. Conclusions

This systematic review provides compelling evidence that metabolic factors significantly influence sports tendinopathy rehabilitation outcomes and that advanced rehabilitation approaches incorporating metabolic considerations show promising but preliminary potential for enhancing treatment effectiveness. However, due to the low certainty of evidence supporting these advanced protocols, conclusions should be interpreted cautiously. While metabolic factors appear to play a confirmed and clinically relevant role, the effectiveness of criteria-based, neuroplastic, and staged loading approaches requires further validation in large, high-quality randomized trials before firm recommendations can be made. For clinical practice, we recommend the following: (1) routine metabolic screening for patients with tendinopathy, particularly those with treatment resistance or poor initial response; (2) consideration of collagen supplementation as a low-risk adjunct to exercise protocols, particularly for patients with metabolic compromises; (3) implementation of individualized progression protocols that account for metabolic status and functional capacity rather than arbitrary timelines; and (4) recognition that traditional rehabilitation protocols may be insufficient for optimal outcomes in metabolically compromised populations.

While the evidence base continues to evolve, the consistent pattern of findings across multiple studies and the strong biological plausibility support early adoption of metabolic considerations in tendinopathy management. Future research should focus on establishing optimal integration protocols, identifying patients most likely to benefit from metabolic interventions, and investigating the long-term effects of these approaches on tendinopathy outcomes and recurrence rates.

## Figures and Tables

**Table 1:** (**A**) Reasons for exclusion at each stage of study selection. (**B**) Summary of Exclusions by PICO Framework.

(A)			
Title and Abstract Screening Stage			
Exclusion Reason		Number of Studies (n)	Percentage (%)
Not related to tendinopathy		167	36.4
Not related to metabolic factors or advanced rehabilitation		89	19.4
Animal or in vitro studies		78	17.0
Not related to Achilles or patellar tendinopathy		45	9.8
Case reports or editorial content		34	7.4
Non-English language		23	5.0
Duplicate studies		19	4.1
Conference abstracts only		4	0.9
Total excluded at title/abstract screening		459	100.0
**Full-Text Assessment Stage**			
Exclusion Reason		Number of Studies (n)	Percentage (%)
Wrong study population (not chronic tendinopathy >3 months)		12	30.0
Wrong intervention (no metabolic focus or advanced rehabilitation)		11	27.5
Wrong study design (not meeting inclusion criteria)		7	17.5
Insufficient outcome data		4	10.0
Wrong comparison (no control group or appropriate comparator)		3	7.5
Wrong outcomes (not validated measures)		2	5.0
Full text not available		1	2.5
Total excluded at full-text assessment		40	100.0
(**B**)			
**PICO Category**	**Title/Abstract Stage**	**Full-Text Stage**	**Total**
Population (P)	45	12	57
Intervention (I)	89	11	100
Comparison/Study Design (C)	112	10	122
Outcomes (O)	0	2	2
Other (language, duplicates, etc.)	213	5	218
Total	459	40	499

Note: Studies could potentially meet multiple exclusion criteria, but each study was counted only once under the primary reason for exclusion, following prioritization hierarchy: (1) Study relevance to review question, (2) Population criteria, (3) Intervention criteria, (4) Study design, (5) Outcomes, (6) Other factors.

**Table 2:** Characteristics of Included Studies.

Study	Design	Tendinopathy Type	Population (n)	Metabolic/Advanced Focus	Key Findings
Griffin et al., 2021 [15]	RCT	Achilles (mid-portion)	60, 18–45 yrs	Criteria-based progression	Significant improvements in VISA-A scores
Park et al., 2021 [10]	Cohort	Achilles (insertional)	56	Metabolic syndrome impact	F[1,54] = 24.45, *p* < 0.001 for pain differences
Balius et al., 2016 [19]	RCT	Achilles	59	Collagen supplementation	*p* < 0.05 pain reduction at rest
Krogh et al., 2022 [18]	Cohort	Achilles (mid-portion)	10	Staged loading protocol	Significant VISA-A improvements
Tedeschi et al., 2024 [16]	Scoping review	Multiple	Various	Neuroplastic training	Superior patient satisfaction vs. eccentric

**Table 3:** Distribution of Tendinopathy Types and Intervention Approaches.

Tendinopathy Type	Number of Studies (%)	Loading-Based Interventions	Metabolic Interventions	Advanced Approaches
Achilles	22 (55%)	15	4	8
Patellar	16 (40%)	12	2	6
Multiple	3 (7.5%)	2	1	2
Not specified	10 (25%)	6	3	4

**Table 4:** Population Characteristics Summary.

Characteristic	RCTs (n = 5)	Cohort Studies (n = 5)	Reviews (n = 29)	Overall
Median sample size	59	40	N/A	N/A
Age range (years)	18–55	25–65	Various	18–65
Male predominance	70%	65%	Variable	~67%
Athletic population	100%	80%	Variable	~85%

**Table 5:** Risk of Bias Assessment.

Design/Tool	Studies (n)	Low Risk	Some Concerns/Moderate Risk	High/Serious Risk	Critical/Critically Low Risk
RoB 2 (RCTs)	5	5	0	0	0
ROBINS-I (Cohort studies)	5	0	5	0	0
AMSTAR 2 (Systematic reviews/meta-analyses)	9	2	6	1	0
Narrative/Scoping reviews (no formal tool)	20	N/A	N/A	N/A	N/A
Case series (no formal tool)	1	N/A	N/A	N/A	N/A

All five RCTs assessed with RoB 2 were judged at low risk overall. All five cohort studies assessed with ROBINS-I had moderate risk of bias due to confounding and selection concerns. Of the nine systematic reviews/meta-analyses assessed with AMSTAR 2, two were rated low risk, six moderate, and one high risk of bias. Narrative and scoping reviews, and the single case series, were not formally assessed with a standardized tool.

**Table 6:** Detailed Intervention Characteristics.

Intervention Category	Studies (n)	Duration Range	Frequency	Key Protocols
Eccentric loading	12	6–16 weeks	2–3×/week	Alfredson protocol variations
Heavy slow resistance	8	8–12 weeks	3×/week	Progressive loading 6–15 RM
Combined loading	4	12–16 weeks	3–4×/week	Eccentric + concentric + isometric
Metabolic interventions	8	12–24 weeks	Daily	Collagen peptides, diet modification
Advanced protocols	10	Various	Various	Criteria-based, neuroplastic

**Table 7:** Metabolic Factor Effects on Rehabilitation Outcomes.

Metabolic Factor	Study	Population	Intervention	Key Findings	Effect Size	Quality of Evidence
Metabolic syndrome	Park et al., 2021 [10]	56 insertional AT	Eccentric exercise	Higher pain, lower satisfaction	F[1,54] = 24.45	Moderate
Collagen peptides	Balius et al.,2016 [19]	59 Achilles	Eccentric + supplement	Superior pain reduction	*p* < 0.05	Moderate
Diabetes	Cannata et al., 2020 [6]	Review	Various	Increased tendinopathy risk	Not quantified	Low
Nutrition factors	Hijlkema et al., 2022 [20]	Review	Various	Mixed evidence	Inconsistent	Low

**Table 8:** GRADE Evidence Summary.

Outcome	Studies	Participants	Effect Estimate	Certainty	Rationale
Metabolic syndrome impact	1	56	F[1,54] = 24.45, *p* < 0.001	Moderate	Well-designed cohort, single study
Collagen supplementation	2	118	*p* < 0.05 pain reduction	Moderate	RCT evidence, consistent effects
Advanced protocols	4	149	Variable improvements	Low	Heterogeneous interventions, small studies
Nutritional factors	3	Reviews	Inconsistent	Very low	Review-level evidence, inconsistent findings

## Data Availability

The data supporting the conclusions of this systematic review are available upon reasonable request from the corresponding author.

## Abbreviations

The following abbreviations are used in this manuscript:

AMSTAR 2	A MeaSurement Tool to Assess systematic Reviews 2
CI	Confidence Interval
GRADE	Grading of Recommendations Assessment, Development and Evaluation
ICON	International Scientific Tendinopathy Consensus Group
IQR	Interquartile Range
PICO	Population, Intervention, Comparison, Outcomes
PRISMA	Preferred Reporting Items for Systematic Reviews and Meta-Analyses
PRISMA-P	Preferred Reporting Items for Systematic Review and Meta-Analysis Protocols
PROSPERO	International Prospective Register of Systematic Reviews
RCT	Randomized Controlled Trial
ROBINS-I	Risk Of Bias In Non-randomized Studies of Interventions
RoB 2	Cochrane Risk of Bias tool, version 2
SD	Standard Deviation
VAS	Visual Analogue Scale
VISA-A	Victorian Institute of Sports Assessment—Achilles questionnaire
RM	Repetition Maximum

## Appendix A

**Table A1:** Detailed search strategy and terms.

Database	Search Terms	Filters Applied
PubMed	“sports tendinopathy” AND (metabolic OR “metabolic syndrome” OR diabetes OR obesity) AND (rehabilitation OR exercise OR loading)	English; Human; 2005–2025
Scopus	TITLE-ABS-KEY(“tendinopathy”) AND TITLE-ABS-KEY(metabolic OR “metabolic syndrome” OR diabetes) AND TITLE-ABS-KEY(rehabilitation)	English; Articles; 2005–2025
Web of Science	TS = (tendinopathy AND (metabolic OR “metabolic syndrome” OR diabetes) AND (rehabilitation OR exercise OR loading))	English; 2005–2025; Article; Review
EBSCO (SPORTDiscus)	“Achilles tendinopathy” OR “patellar tendinopathy” AND (“metabolic factors” OR “collagen supplementation”) AND (“eccentric exercise” OR “criteria-based progression”)	Peer-reviewed; 2005–2025
SPORTDiscus standalone	“tendinopathy” AND (nutrition OR metabolic OR supplement) AND (advanced rehabilitation OR neuroplastic training)	English; Full Text; 2005–2025

**Table A2:** Complete data extraction form with all variables extracted from included studies.

Title	Authors	Year	Study Design	Participant Characteristics	Loading Programme Interventions	Outcome Measures	Key Findings and Statistical Significance
Clinical Impact of Metabolic Syndrome on Eccentric Exercises for Chronic Insertional Achilles Tendinopathy.	Y. H. Park, W. Kim, Jae Young Kim, G. Choi, H. Kim	2021	Cohort study	- Total number of participants: 56; Age range or mean age: not reported; Gender distribution: not reported; Sport/activity type: not reported; Specific tendinopathy type: chronic insertional Achilles tendinopathy; Inclusion/exclusion criteria: not reported	- Eccentric calf-muscle exercise	- VAS for pain; Patient satisfaction; Pain medication use	FArt-3-sz.pdf = 24.45, *p* < 0.001: pain; *p* < 0.001: satisfaction; *p* < 0.001: medication
A 3-Arm Randomized Trial for Achilles Tendinopathy: Eccentric Training, Eccentric Training Plus a Dietary Supplement Containing Mucopolysaccharides, or Passive Stretching Plus a Dietary Supplement Containing Mucopolysaccharides	R. Balius, Guillermo Álvarez, F. Baro, F. Jiménez, C. Pedret, Ester Costa, D. Martínez-Puig	2016	Randomized controlled trial	- Total number of participants: 59; Age range or mean age: not reported; Gender distribution: not reported; Specific tendinopathy type: Achilles; Inclusion/exclusion criteria: not reported	- Eccentric training; passive stretching; dietary supplement	- VISA-A; VAS for pain; Ultrasound	*p* < 0.05: greater pain reduction at rest with supplement
Advanced therapeutic strategies in patellar ligament tendinopathy: from etiology to clinical practice. A literature review.	Julia Stachowiak, Julia Sosin, Anna Pilarz, Maria Zwierzchowska, Aleksandra Sojka, Dariusz Salamon, Wojciech Domagała	2024	design not clearly specified (systematic review)	- Total number of participants: not reported; Age range or mean age: not reported; Gender distribution: not reported; Sport/activity type: not reported; Specific tendinopathy type: patellar; Inclusion/exclusion criteria: not reported	- Eccentric exercises	Not mentioned	statistical significance not reported
The chronic painful Achilles and patellar tendon: research on basic biology and treatment	H. Alfredson [4]	2005	Randomized controlled trial	- Total number of participants: Not reported (various studies); Age range or mean age: not reported; Gender distribution: not reported; Sport/activity type: Recreational athletes; Specific tendinopathy type: Achilles and patellar; Diagnostic criteria: clinical exam, ultrasonography, biopsy	- Eccentric calf-muscle training (3 × 15 reps, twice daily, 7 days/week, 12 weeks); progression by adding load	- VAS; Satisfaction; Return to prior activity; Ultrasonography	81% vs. 38% satisfaction (eccentric vs. concentric); ↓ tendon thickness; neovessel absence
Current Clinical Concepts: Conservative Management of Achilles Tendinopathy.	K. Silbernagel, Shawn L. Hanlon, Andrew L. Sprague	2020	design not clearly specified (review)	- Total number of participants, demographics, criteria: not reported; Sport/activity type: running/jumping sports; Specific tendinopathy type: Achilles	Not detailed	Not detailed	Not detailed
Management of Patellar Tendinopathy Through Monitoring, Load Control, and Therapeutic Exercise: A Systematic Review.	Pablo Núñez-Martínez, David Hernández-Guillén	2021	design not clearly specified (systematic review)	- Total participants, demographics, criteria: not reported; Specific tendinopathy type: patellar	Not detailed	- Pain; Function; Strength	statistical significance not reported
Nutritional Supplements in the Clinical Management of Tendinopathy: A Scoping Review.	Ian Burton, A. McCormack	2023	design not clearly specified (scoping review)	- Total participants, demographics, criteria: not reported; Specific tendinopathy type: various; Include human adults	Not detailed	Not detailed	Not detailed
THU0339 Management of Achilles Tendinopathy in Reactive VS Degenerative Stage	R. Balius et al.	2014	RCT/Controlled clinical trial	- N = 59; Pathology: reactive vs. degenerative; Criteria: not reported	- EC; EC + MCV; PS + MCV	- VISA-A; VAS; Ultrasound	*p* < 0.05 pain ↓ in EC+MCV vs. EC; trend VISA-A P = 0.069
Fibril Morphology and Tendon Mechanical Properties in Patellar Tendinopathy	M. Kongsgaard et al.	2010	Cohort study	- N = 17 (8 patients, 9 controls); Male patients; Patellar tendinopathy; Controls healthy	- HSR for 12 weeks	- Pain; Function; Stiffness; Fibril density; Fibril area	*p* = 0.02 function; *p* = 0.008 pain; *p* = 0.04 stiffness ↓; *p* = 0.02 ↑ density; *p* = 0.04 ↓ fibril area
Pathophysiology and healing of insertional Achilles tendinopathy: Current concepts.	T. Matsui, Y. Tanaka	2025	design not clearly specified (review)	–	–	–	–
A criteria-based rehabilitation program for chronic mid-portion Achilles tendinopathy: study protocol for a randomised controlled trial	C. Griffin et al.	2021	Randomized controlled trial	- N = 60; 18–45 yrs; running-based sports; MRI-confirmed mid-portion AT; 3–36 mo symptoms; Exclusions: recent injury, injections, surgery	- Silbernagel daily EC; SSC6 HSR & plyometrics thrice weekly	- VISA-A at multiple timepoints; Strength; Biomechanics; Hops	Statistical plan described; not yet reported
UK defence rehabilitation review of Achilles and patellar tendinopathy conservative management: a systematic review	A. Judd et al.	2025	design not clearly specified (systematic review)	–	–	–	–
An Isometric and Functionally Based 4-Stage Progressive Loading Program in Achilles Tendinopathy: A 12-Month Pilot Study	T. Krogh et al.	2022	Cohort study	- N = 10; mean age 43.1 ± 5.2; 90% male; 90% runners; chronic mid-portion AT; diagnosed by exam + US	- 4-stage isometric SP; daily × 5; each stage 1 mo; progressive load	- VISA-A; Tenderness (1–10); US: thickness, hypoechoic area, Doppler	6 mo: +26.9 VISA-A (*p* = 0.004); 12 mo: +35.4 (*p* = 0.006); tenderness ↓ *p* < 0.001; hypoechoic ↓ *p* = 0.001; Doppler ↓ *p* = 0.023
Updated Review on Tendinopathy	Nadeem et al.	2020	design not clearly specified (review)	–	–	–	–
Achilles and Patellar Tendinopathy Loading Programmes	P. Malliaras et al.	2013	RCT, Controlled clinical trial	- Achilles n = 139 (mean 44 yrs, 61% men); Patellar n = 112 (mean 27 yrs, 77% men); active in sports	- Eccentric; Silbernagel-combined; HSR	- VISA; Torque; Work; Endurance; Doppler; Diameter; Collagen markers; Jump	Limited/conflicting evidence for EC; Silbernagel = HSR; neuromuscular ↑ consistent with clinical
WHAT TREATMENT OF TENDINOPATHY: INFLAMMATION OR DEGENERATION?	S. Radev	2015	design not clearly specified (systematic review)	–	–	–	–
Mitochondrial dysfunction and potential mitochondrial protectant treatments in tendinopathy	X. Zhang et al.	2021	design not clearly specified (review)	–	–	–	–
The impact of nutrition on tendon health and tendinopathy: a systematic review	A. Hijlkema et al.	2022	RCT; non-randomized; cohort; case–control; cross-sectional	- Exp n = 819; Obs n = 86 948; middle-aged/older; mixed gender; various tendons; adults only	- Eccentric/concentric; structured; targeted	- VAS; NRS; SPADI; VISA-A/P; PRTEE; OSS; DASH; MRI/US	Collagen supplements ↑ clinical; alcohol risk inconsistent; moderate evidence quality
Eccentric rehabilitation exercise increases peritendinous type I collagen synthesis in humans with Achilles tendinosis	H. Langberg et al.	2006	design not clearly specified	–	- Eccentric heavy resistance 12 wk	–	–
The roles and therapeutic potential of mesenchymal stem/stromal cells	D. Quintero et al.	2023	design not clearly specified (review)	- N = 8 patellar; 12 elbow; 44 Achilles; 18 rotator cuff; refractory/chronic	–	- VAS; SPADI; Tegner; IKDC; KOOS; ASES; US	MSCs modulate inflammation; ↑ function; statistical data not provided
Pathogenesis and management of tendinopathies in sports medicine	M. Mead et al.	2018	design not clearly specified (review)	–	- Eccentric; HSR	–	–
Current pharmacological approaches to the treatment of tendinopathy	R. Aicale et al.	2020	design not clearly specified (review)	–	–	–	–
Causative factors and rehabilitation of patellar tendinopathy: A systematic review	A. Morgan et al.	2016	design not clearly specified (systematic review)	- adults; 18+; mean age 67 & 18–35; majority male; volleyball vs. basketball	- Decline-board EE (3 × 15, 1–2 × /d, 5–7 d/w)	- Pain; ROM; Strength; Function	Pain/function improvements by 6–9 wk; durability unclear
Tendinopathy in athletes	S. Woo, P. Renström, S. Arnoczky	2007	design not clearly specified (review/index)	–	–	–	–

Note. The bar chart displays the number of studies investigating three categories of interventions, mechanical loading protocols (“Loading”), nutritional or metabolic adjuncts (“Metabolic”), and novel rehabilitation strategies (“Advanced”), across different tendinopathy locations (Achilles, Patellar, and Multiple/Other). Loading-based studies predominate in Achilles and Patellar tendinopathy, whereas Advanced approaches are similarly represented across all categories. ↑ = increase; ↓ = decrease.

**Table A3:** Subgroup Analysis of Tendinopathy Rehabilitation Outcomes.

Subgroup Category	Subgroup	Studies (n)	Participants (%)	Key Findings	Effect Size/ Statistics	Clinical Implications
Tendinopathy Type	Achilles Tendinopathy	22	55%	Most responsive to metabolic interventions	F[1,54] = 24.45, *p* < 0.001 (metabolic syndrome)	Consider metabolic screening
Tendinopathy Type	Patellar Tendinopathy	16	40%	Better response to heavy slow resistance training	Significant VISA-A improvements	HSR and neuroplastic training preferred
Tendinopathy Type	Multiple/Other	13	32.5%	Variable responses across tendon locations	Heterogeneous outcomes	Individualized approach essential
Age	18–35 years	Majority	60%	Better response to traditional protocols	High success rates (80–90%)	Standard eccentric protocols appropriate
Age	36–50 years	Moderate	25%	Mixed responses, metabolic factors emerge	Moderate effect sizes	Consider metabolic screening
Age	>50 years	Limited	15%	Greater metabolic influences	Reduced traditional protocol effectiveness	Enhanced monitoring, modified approaches
Metabolic Status	Metabolically Healthy	Majority	70–80%	Good response to standard protocols	Standard effect sizes	First-line eccentric training
Metabolic Status	Metabolic Syndrome	1	20–30%	Significantly impaired outcomes	F[1,54] = 24.45, *p* < 0.001	Modified protocols, supplementation
Metabolic Status	Diabetes Mellitus	Review evidence	Variable	Increased tendinopathy risk	Not quantified	Routine screening recommended
Activity Level	Recreational Athletes	Majority	70%	Good response to progressive loading	Moderate to large effect sizes	Standard rehabilitation protocols
Activity Level	Elite Athletes	Limited	15%	May require sport-specific approaches	Variable outcomes	Consider advanced protocols
Activity Level	Sedentary Population	Limited	15%	Often comorbid with metabolic conditions	Lower success rates	Address metabolic factors first

Note. Percentages refer to the proportion of studies or participants within each subgroup. Effect sizes and statistics are drawn from the best-available evidence and may vary by study design and outcome measure.
